# Zinc-glutathione in Chinese Baijiu prevents alcohol-associated liver injury

**DOI:** 10.1016/j.heliyon.2023.e13722

**Published:** 2023-02-13

**Authors:** Yinrui Feng, Wenrui Liu, Te Ba, Zhenghui Luo, Yuan Ma, Guotao Tang, Y. James Kang

**Affiliations:** a Regenerative Medicine Research Center, Sichuan University West China Hospital, Chengdu, Sichuan, 610041, China; b Tennessee Institute of Regenerative Medicine, University of Tennessee Health Science Center, Memphis, TN, 38163, USA

**Keywords:** Zinc-glutathione, Chinese Baijiu, Alcohol, Alcoholism, Alcohol metabolism, Liver injury

## Abstract

Zinc depletion is associated with alcohol-associated liver injury. We tested the hypothesis that increasing zinc availability along with alcohol consumption prevents alcohol-associated liver injury. Zinc-glutathione (ZnGSH) was synthesized and directly added to Chinese Baijiu. Mice were administered a single gastric dose of 6 g/kg ethanol in Chinese Baijiu with or without ZnGSH. ZnGSH in Chinese Baijiu did not change the likeness of the drinkers but significantly reduced the recovery time from drunkenness along with elimination of high-dose mortality. ZnGSH in Chinese Baijiu decreased serum AST and ALT, suppressed steatosis and necrosis, and increased zinc and GSH concentrations in the liver. It also increased alcohol dehydrogenase and aldehyde dehydrogenase in the liver, stomach, and intestine and reduced acetaldehyde in the liver. Thus, ZnGSH in Chinese Baijiu prevents alcohol-associated liver injury by increasing alcohol metabolism timely with alcohol consumption, providing an alternative approach to the management of alcohol-associated drinking.

## Abbreviations

ZnGSH: Zinc-glutathione
GSH: Glutathione
ALD: Alcohol-associated liver disease
AST: Aspartate transaminase
ALT: Alanine transaminase
TG: Triglyceride
ADH: Alcohol dehydrogenase
ALDH: aldehyde dehydrogenase
GC‒MS: Gas chromatography‒mass spectrometry
GDA: Generic descriptive analysis
OCT: Cutting temperature gel
H&E: Hematoxylin and eosin

## 1 Introduction

Alcohol has been an important part of human culture for thousands of years. Excessive alcohol consumption is a long-standing problem for human civilization, and alcohol abuse is a serious global health problem with a significant socioeconomic burden in most countries [1]. Abstaining from alcoholic drinking would be an effective solution for all of the problems brought by alcohol consumption, but it would not become a reality.

The liver is the primary metabolic organ for alcohol [2]. Alcohol dehydrogenase is the first enzyme in the cytosol of hepatocytes to catalyze the reaction breaking down alcohol to aldehyde, which in turn is degraded to carbon dioxide and water by the enzyme aldehyde dehydrogenase, being localized in mitochondria [3]. A complete metabolism of alcohol in the liver is related not only to the liver injury but also to alcohol-associated injuries to multiple organ systems [4]. Zinc is the essential cofactor for the two enzymes involved in alcohol metabolism [5]. Therefore, the availability of zinc in different subcellular locations to meet the requirement for alcohol metabolism critically affects the pathogenesis of alcohol-associated injury to the liver and other organs [4].

It has been shown that binge drinking of ethanol results in a rapid decline in hepatic zinc stores, particularly in the liver mitochondria [6]. Zinc deficiency is one of the most consistent nutritional/biochemical disorders in alcohol-associated liver disease [7]. Consequently, zinc supplementation attenuates ethanol-induced liver injury and enhances liver regeneration in murine models [4,5,[8], [9], [10], [11]].

Efforts have been made to extrapolate the experimental results to human clinical applications using zinc as a therapeutic agent to treat alcohol-associated liver disease [12,13]. Although these efforts have been shown to be promising, their effectiveness is jeopardized by the continuity of liver injury from constant alcohol consumption. Therefore, if zinc supplementation can be managed in conjunction with alcohol consumption to prevent liver injury, it would be a better approach than the treatment for liver disease developed after alcohol consumption.

Depletion and altered subcellular distribution of zinc along with alcohol consumption is a critical issue for alcohol-associated liver injury [6]. Therefore, we propose that if zinc availability can be ensured along with alcohol consumption, alcohol-associated liver injury would be prevented. Therefore, we attempted to add a zinc compound to alcoholic beverages, ensuring that the addition of the zinc compound does not change any of the alcohol-associated drinking properties, including the color, aroma, taste, and proof.

To fulfill the goal of maintaining the likeliness of a drinker for a particular alcoholic beverage but preventing alcohol-associated liver injury, we made a zinc-glutathione (ZnGSH) compound for zinc supplementation in a popular Chinese liquor, Chinese Baijiu. GSH is not only able to form a complex with zinc but itself also has antioxidant capacity. We hypothesize that ZnGSH would be delivered to the liver along with the alcoholic beverage, preventing zinc depletion and ensuring the activity of the two alcohol metabolic enzymes in the liver.

In the present study, we used a mouse model of binge drinking to determine the protective effect of ZnGSH in Chinese Baijiu on alcohol-associated liver injury. The results proved that the addition of ZnGSH to the Chinese liquor indeed prevented alcohol-associated liver injury.

## 2 Materials and methods

### 2.1 Materials

Chinese Baijiu was produced by Guizhou Zhukang District (Guizhou, China). The metal salt Zn(SO)_4_·7H2O and other reagents were of analytical grade and purchased from Kelong (Chengdu, China). Glutathione was purchased from Adamas-beta (Shanghai, China). Commercial assay kits for serum alanine aminotransferase (ALT), aspartate aminotransferase (AST), aldehyde dehydrogenase (ALDH), alcohol dehydrogenase (ADH), triglyceride (TG), glutathione (GSH) and blood ethanol were obtained from Jiancheng Institute of Biotechnology (Nanjing, China). BCA protein assay kits were obtained from Thermo Fisher (USA). Bovine serum albumin (BSA) was purchased from Gentihold (Beijing, China). Acetaldehyde assay kit was purchased from Megazyme (Bray, Wicklow, Ireland). Endotoxin ELISA kit was obtained from Bioswamp (Wuhan, China). F4/80 (A1256) and TNF-α ELISA kits were obtained from ABclonal Technology (Wuhan, China). Oil red O was purchased from Solarbio Life Sciences (G1260, Beijing, China). Cleaved Caspase 3 (9661) and HRP-labeled secondary antibody (8114) were obtained from Cell Signaling Technology (Beverly, Ma, USA). Diaminobenzidine (DAB-2031) was purchased from MXB Biotechnologies (Fuzhou, China).

### 2.2 Synthesis and characterization of ZnGSH

Zinc-glutathione (ZnGSH) was synthesized as follow: ZnSO_4_ was mixed with GSH at a 1:2 M ratio in 50% ethanol. The reaction mixture was stirred at 25 °C for 10 h. Then, the fraction was concentrated by rotary evaporation. ZnGSH was recrystallized after condensation, followed by characterization by Fourier transform infrared spectrometry (Nicolet 6700, Thermo Fisher) and LC–MS (1260–6110, Agilent).

### 2.3 Preparation and characterization of Chinese Baijiu containing ZnGSH

ZnGSH was directly added to Chinese Baijiu at final concentrations of 75 mg Zn and 715 mg GSH/L. After full striation, the liquor containing ZnGSH was stored at room temperature overnight. The final product, Chinese Baijiu containing ZnGSH, was sampled for chemical and sensory analysis according to GB/T 26760. Eight parameters, including alcoholic strength, acetic acid, ethyl acetate, ethyl hexanoate, solid content, methyl alcohol, cyanide, and plumbum, were detected by Guizhou High-tech Testing Co., Ltd. (Guizhou, China), a professional third-party testing institute. All the detection methods refer to GB/T 10345.

Generic descriptive analysis (GDA) was carried out using 17 trained assessors (five women and twelve men) with at least forty years of experience in Chinese Baijiu. The sensory profile of the fractions was performed across four sessions, referring to GB/T 26760 sensory methodology (Table 1). The final report proved that there was no difference between the original Chinese Baijiu and the ZnGSH containing Chinese Baijiu.

**Table 1:** Average concentrations of aroma compounds in Chinese Baijiu and Chinese Baijiu + ZnGSH.

Aroma compounds	Baijiu	Baijiu + ZnGSH	Scoring *criteria*
Ethanol %vol	53.6	53.0	/
Total acid (acetic acid) (g/L)	1.76	2.36	≥1.40
Total acetate (ethyl acetate) (g/L)	3.89	3.21	≥2.20
Ethyl hexanoate (g/L)	0.01	0.03	≤0.30
Solid (g/L)	0.01	0.25	≤0.70
Methyl alcohol (g/L)	0.20	0.20	≤8.00
HCN (mg/L)	0.05	0.12	≤0.60
Pb (mg/kg)	<0.04	<0.04	≤0.50

Baijiu: Chinese Baijiu; Baijiu + ZnGSH: Chinese Baijiu containing ZnGSH.

### 2.4 Animals

All procedures for animal care and experimental operation were approved by the Institutional Animal Care and Use Committee (IACUC) of Sichuan University West China Hospital following the guidelines of the US National Institutes of Health (approval no. 20220124003A). Male C57BL/6 mice (8–12 weeks old, weighing 18–25 g) were obtained from the Ensiweier Experimental Animal Breeding and Research Center (Chongqing, China). Mice were housed in standard cages at a controlled temperature of 22 ± 1 °C, humidity of 50% and a 12:12-h dark-light cycle. All mice were given *ad libitum* access to standard chow and deionized water.

### 2.5 Acute ethanol challenge

A binge drinking mouse model developed by Carson and Pruett [15] was followed for ethanol challenge, with minor modifications. This model was designed to achieve blood alcohol levels that would produce physiologically abnormal effects comparable to human binge drinking. Animals were divided into four treatment groups (seven mice per group) in a 2 × 2 factorial design ( ± zinc-glutathione, ± Chinese Baijiu): 1) isocaloric maltose water control, 2) Chinese Baijiu, 3) ZnGSH in isocaloric maltose water, and 4) Chinese Baijiu containing ZnGSH. Mice in groups 2 and 4 were administered Chinese Baijiu or Chinese Baijiu containing ZnGSH in a single alcohol dose of 6 g/kg body weight by gavage, and groups 1 and 3 mice received isocaloric maltose water without (group 1) or with (group 3) ZnGSH by the same route on the same schedule. Chinese Baijiu containing ZnGSH contained 75 mg Zn element/L. Experimental animals were sacrificed, and plasma, liver, and gastrointestinal tissue samples were collected at 12, 24, and 48 h after alcohol administration. The collected samples were either preserved at −80 °C for Zn concentration determination or embedded in optimal cutting temperature gel (OCT, Leica, Germany) or paraffin for histological analyses.

### 2.6 Behavior evaluation

EtOH-induced behavioral effects were evaluated using criteria published previously [15], with a slight modification to show the time to obtain sobriety with mice. Stages of inebriation were assigned the following numerical values: no effect = 1; mild ataxia (spontaneous locomotion, slight posture change, but abdomen still elevated above follow-up of the cage) = 2; severe ataxia (little if any spontaneous locomotion, pronounced staggering, abdomen in contact with follow-up of cage during movement) = 3, loss of righting reflex = 4. Evaluations were performed blindly.

### 2.7 Serum enzymes assay

Blood samples were obtained from the orbital varix before the mice were sacrificed. Serum alanine aminotransferase (ALT) and aspartate aminotransferase (AST) were measured using commercially available kits following the procedure described previously [16].

### 2.8 Histopathological examination

Liver and intestinal histological slides were prepared as described previously [17], and hematoxylin and eosin (H&E) staining of the tissue sections was observed by light microscopy.

### 2.9 Determination of hepatic lipid deposition

Hepatic lipid deposition was assessed by histochemical detection of neutral lipids. Liver tissues were frozen in Tissue-OCT compound, and cryostat tissue sections were cut at 10 mm, fixed with 4% paraformaldehyde for 15 min, and stained with an oil red O procedure. A staining working solution was prepared by diluting the saturated oil red O stock solution with deionized water (3:2). The final solution was passed through 0.2-mm filter paper before use. The polymethyl-fixed tissue samples were placed in the working solution in an oven at 45 °C for 10 min followed by washing with 60% isopropanol to remove nonspecific staining. The area of lipid deposition was measured using the image analyzer ImageJ, and the ratio of lipid deposition to the entire surface area of the liver was calculated. Hepatic triglyceride (TG) was measured by a commercial kit following the manufacturer's instructions.

### 2.10 Immunohistochemistry staining

Sections of liver were deparaffinized in easy tissue clearant and rehydrated in gradient alcohol. The sections were blocked with 3% hydrogen peroxide at room temperature for 20 min under dark conditions, followed by incubation with 2% bovine serum albumin at 37 °C for 1 h. Then, the tissue sections were incubated overnight with primary antibodies (rat anti-F4/80, 1:50; rat anti-cleaved caspase 3, 1:100) at 4 °C and subsequently incubated with HRP-labeled goat anti-rabbit secondary antibody at 37 °C for 1 h. Finally, the sections were processed with a freshly mixed diaminobenzidine kit according to the manufacturer's instructions, and the nuclei were counterstained with hematoxylin. Three to five visual fields of each sample were randomly selected and collected with a microscope (Eclipse 80i, Nikon) and analyzed by ImageJ software.

### 2.11 Assay of endotoxin and TNF-α

Levels of plasma endotoxin and TNF-α were determined using commercial ELISA kits according to the manufacturer's manuals.

### 2.12 Assay of ethanol and acetaldehyde

Blood samples were obtained from the orbital varix before the mice were sacrificed. Liver segments were separated from fresh tissue, as previously reported [11]. Plasma and hepatic ethanol and acetaldehyde contents were determined according to the supplier's protocol.

### 2.13 Assays of alcohol dehydrogenase and aldehyde dehydrogenase activities

Liver and gastrointestinal segments were separated from fresh tissue, as previously reported [11]. The BCA protein quantification assay kit was used to measure the protein contents in tissues. Alcohol dehydrogenase (ADH) and aldehyde dehydrogenase (ALDH) activities were measured by a commercial kit following the manufacturer's instructions.

### 2.14 Assay of glutathione

The BCA protein quantification assay kit was used to measure the protein contents in tissues. Glutathione (GSH) was measured by a commercial kit according to the manufacturer's instructions.

### 2.15 Zinc concentrations in the liver

Lyophilized liver samples from different hepatic lobes were digested in concentrated nitric acid overnight at 60 °C. Hepatic zinc concentrations were determined by atomic absorption spectrometry (ICE 3500, Thermo Scientific, USA) following the procedure described previously [18]. The amount of zinc was normalized to the dry tissue weight.

### 2.16 Statistical analysis

All data were analyzed using GraphPad Prism 7.0 and are presented as the mean ± SEM. ANOVA was used to compare continuous variables. Comparisons between groups were made using two-way analysis with Tukey's multiple comparisons test. The level of significance was considered at P < 0.05.

## 3 Result

### 3.1 Effects of ZnGSH on time to obtain sobriety, survival, and biomarkers

**Fig. 1:**
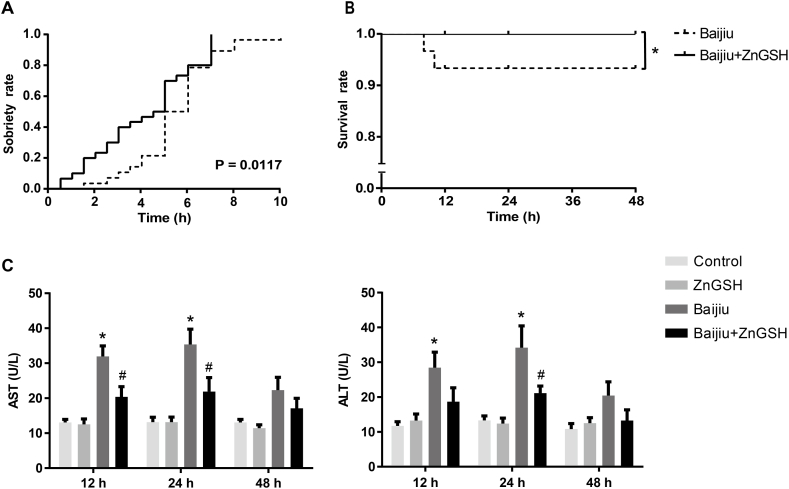
Effects of ZnGSH on time to obtain sobriety, survival and biomarkers. (A) Time to obtain sobriety and (B) survival rate of mice (Chinese Baijiu: n = 30, Chinese Baijiu-ZnGSH: n = 30). (C) Serum AST and ALT enzyme activities. Control: isocaloric maltose water; ZnGSH: isocaloric maltose water containing ZnGSH; Baijiu: Chinese Baijiu (6 g/kg BW); Baijiu + ZnGSH: Chinese Baijiu containing ZnGSH (6 g/kg BW). Data are expressed as the mean ± standard error (SEM), n = 6 for each group. * Significantly different from the Control group, and # significantly different from the Control and the Baijiu groups.

The state of time to obtain sobriety was defined from a behavioral score of 4 to 1. Behavioral scores were recorded for each mouse every 10 min until no behavioral effects were evidenced. The behavior of the control mice was used as a reference. As shown in Fig. 1A, the time to obtain sobriety of the mice drinking Chinese Baijiu was 5.5 ± 0.3 h. In contrast, the time of the mice drinking Chinese Baijiu containing ZnGSH was 4.1 ± 0.4 h, being significantly shorter than that spent by the former. As shown in Fig. 1B, two mice died in the Chinese Baijiu drinking group, constituting 6.7% mortality; in contrast, no deaths were found in the group drinking Chinese Baijiu containing ZnGSH. Chinese Baijiu exposure elicited a significant increase in serum AST and ALT levels at 12 and 24 h after dosing, and this effect was significantly suppressed in the mice drinking Chinese Baijiu containing ZnGSH (Fig. 1C).

### 3.2 Effects of ZnGSH on liver and gastrointestinal injuries

**Fig. 2:**
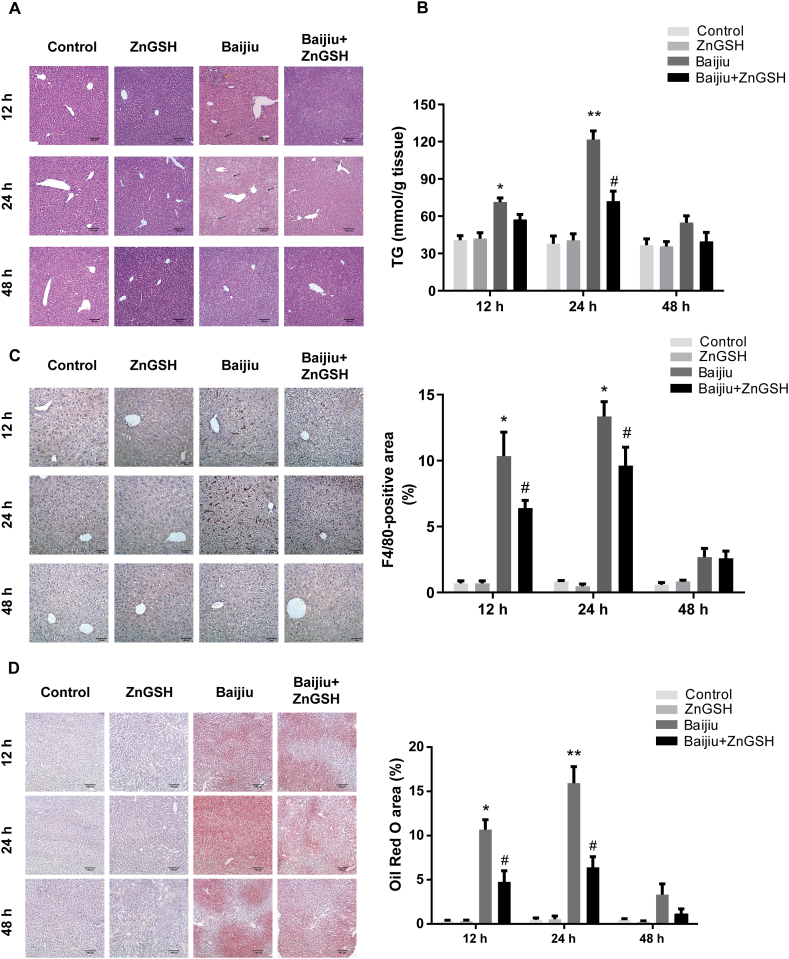
Effects of ZnGSH on liver injury. (A) HE staining of the liver. The yellow arrow indicates inflammatory cell infiltration, and the black arrows indicate swollen hepatocytes. Scale bar = 100 μm for all images. (B) TG in the liver. (C) F4/80-positive cells immunohistochemical staining (brown) in the liver and semiquantitative analysis of the total F4/80-positive cells area. Scale bar = 50 μm. (D) Representative Oil Red O staining in the liver shows deposited lipid droplets and semiquantitative analysis of the total Oil Red O area. Scale bar = 100 μm. Control: isocaloric maltose water; ZnGSH: isocaloric maltose water containing ZnGSH; Baijiu: Chinese Baijiu (6 g/kg BW); Baijiu + ZnGSH: Chinese Baijiu containing ZnGSH (6 g/kg BW). Data are expressed as the mean ± standard error (SEM), n = 6 for each group. * Significantly different from the Control group, ** Significantly from the Baijiu group at 12 h, and # significantly different from the Control and Baijiu groups.

There was an accumulation of small fat droplets (microvesicular steatosis) in hepatocytes, along with severe ballooning, inflammatory cell infiltration and necrosis in the liver of the mice drinking Chinese Baijiu at 12 and 24 h after dosing. Chinese Baijiu containing ZnGSH significantly inhibited steatosis and necrosis in the liver, as seen by the diminished fatty infiltration in hepatocytes (Fig. 2A), as well as a remarkable reduction in inflammatory cell infiltration (Fig. 2C). Further analysis using oil red O staining and triglyceride measurement revealed that Chinese Baijiu containing ZnGSH significantly reduced lipid deposition in the liver in comparison with that caused by Chinese Baijiu alone (Fig. 2B, D).

**Fig. 3:**
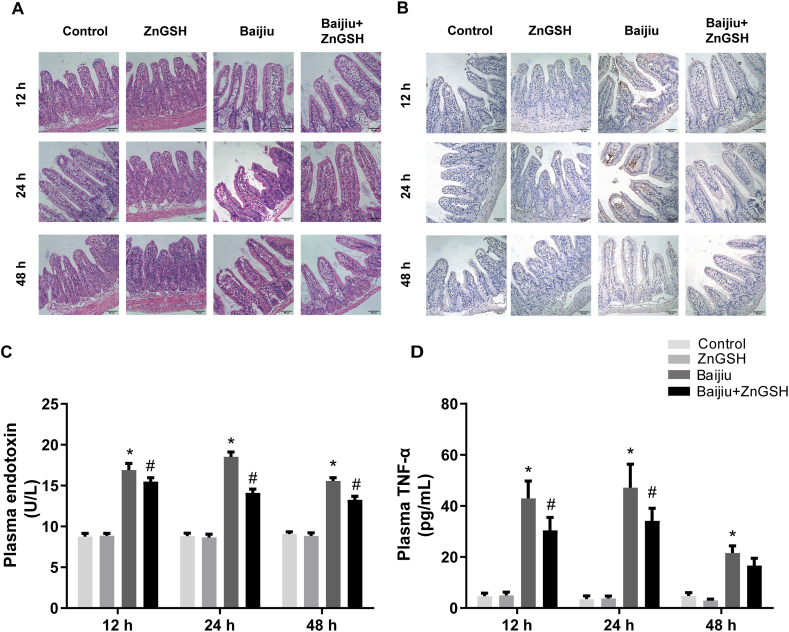
Effects of ZnGSH on intestinal injury. (A) HE staining and (B) Cleaved caspase-3 immunohistochemical staining (brown) in the intestine. Scale bar = 50 μm for all images. (C) Endotoxin and (D) TNF-α in the plasma. Control: isocaloric maltose water; ZnGSH: isocaloric maltose water containing ZnGSH; Baijiu: Chinese Baijiu (6 g/kg BW); Baijiu + ZnGSH: Chinese Baijiu containing ZnGSH (6 g/kg BW). Data are expressed as the mean ± standard error (SEM), n = 6 for each group. * Significantly different from the Control group, and # significantly different from the Control and Baijiu groups.

Chinese Baijiu also caused injury to the mucosal lining of the small intestine along with cellular swelling (Fig. 3A) and cell apoptosis (Fig. 3B) at 12 and 24 h after dosing, as well as a remarkable increase in plasma endotoxin and TNF-α (Fig. 3C and D). These effects were also prevented by ZnGSH in Chinese Baijiu (Fig. 3A–D).

**Fig. 4:**
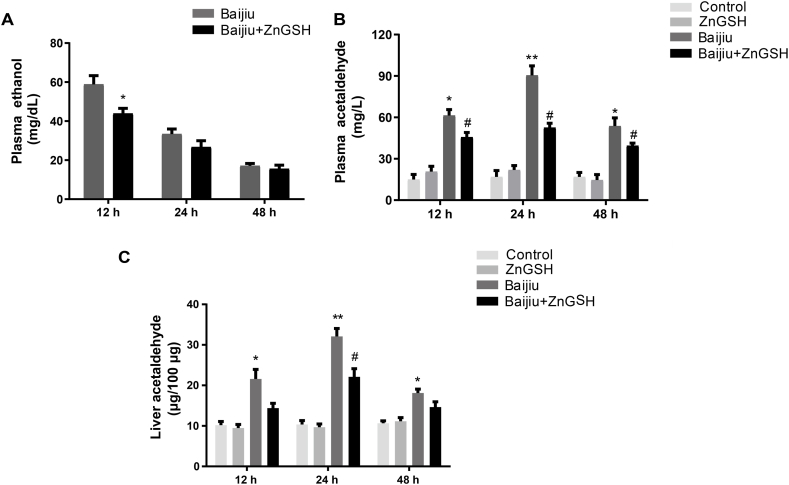
Effect of ZnGSH on alcohol-metabolizing production. (A) Ethanol and (B) acetaldehyde in the plasma. (C) Acetaldehyde in the liver. Control: isocaloric maltose water; ZnGSH: isocaloric maltose water containing ZnGSH; Baijiu: Chinese Baijiu (6 g/kg BW); Baijiu + ZnGSH: Chinese Baijiu containing ZnGSH (6 g/kg BW). Data are expressed as the mean ± standard error (SEM), n = 5 for each group. * Significantly different from the Control group, ** Significantly from the Baijiu group at 12 h, and # significantly different from the Control and Baijiu groups.

Chinese Baijiu caused a significant increase in alcohol (Fig. 4A) and acetaldehyde concentrations in the blood (Fig. 4B) and liver (Fig. 4C) at 12 and 24 h after dosing, and ZnGSH in Chinese Baijiu significantly suppressed this elevation at both time points (Fig. 4A–C).

### 3.3 Effect of ZnGSH on alcohol-metabolizing enzymes

**Fig. 5:**
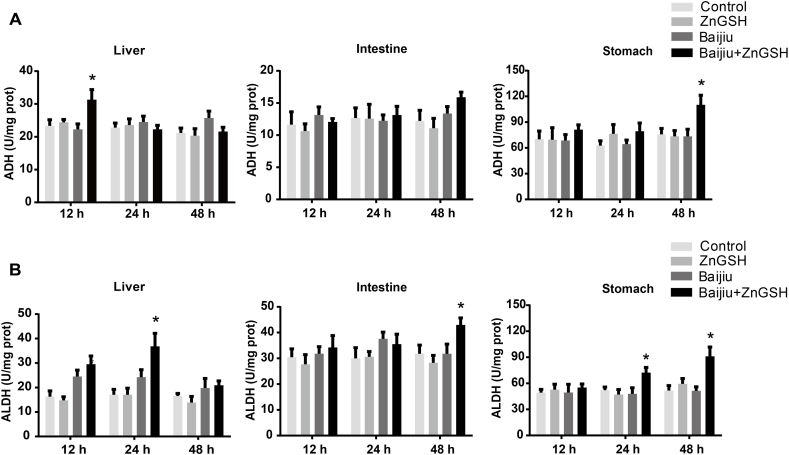
Effect of ZnGSH on alcohol-metabolizing enzymes. (A) ADH in the liver, intestine and stomach. (B) ALDH in the liver, intestine and stomach. Control: isocaloric maltose water; ZnGSH: isocaloric maltose water containing ZnGSH; Baijiu: Chinese Baijiu (6 g/kg BW); Baijiu + ZnGSH: Chinese Baijiu containing ZnGSH (6 g/kg BW). Data are expressed as the mean ± standard error (SEM), n = 6 for each group. * Significantly different from all other groups.

**Fig. 6:**
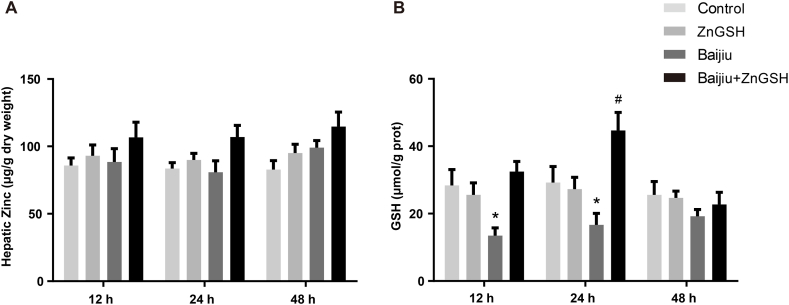
Effect of ZnGSH treatment on zinc and glutathione in the liver. (A) Zinc and (B) glutathione in the liver. Control: isocaloric maltose water; ZnGSH: isocaloric maltose water containing ZnGSH; Baijiu: Chinese Baijiu (6 g/kg BW); Baijiu + ZnGSH: Chinese Baijiu containing ZnGSH (6 g/kg BW). Data are expressed as the mean ± standard error (SEM), n = 5 for each group. * Significantly different from the Control group, and # significantly different from all other groups.

Chinese Baijiu did not alter ADH activities in the liver or in the gastrointestinal tract. In contrast, Chinese Baijiu containing ZnGSH significantly increased ADH activities in the liver at 12 h after dosing and increased the same enzyme activity in the stomach at 48 h after dosing (Fig. 5A). Chinese Baijiu slightly increased ALDH activities only in the liver at 12 h after dosing, but Chinese Baijiu containing ZnGSH boosted the enzyme activity not only at 12 h but also at 24 h in the liver after dosing. In addition, the latter significantly increased ALDH activities in the interstine and stomach at 48 h after dosing (Fig. 5B). In addition, Chinese Baijiu containing ZnGSH increased zinc concentrations in the liver at all three times measured (Fig. 6A). It was also observed that Chinese Baijiu significantly depleted GSH levels in the liver at 12 and 24 h after dosing, and ZnGSH in Chinese Baijiu not only presented this depletion but also significantly elevated GSH concentrations in the liver at 24 h after dosing (Fig. 6B).

## 4 Discussion

Excessive alcohol consumption is considered to be a major cause of liver injury [1]. When excessive amounts of ethanol are consumed, the liver is unable to effectively complete alcohol metabolism [19]. In addition, insufficient zinc availability further worsens the incomplete alcohol metabolism because zinc is the essential cofactor of ADH and ALDH [5]. Zinc supplementation has been reported to prevent alcohol-associated liver injury in mice [9,20,21]. However, it is difficult to achieve the compliance for a drinker to take a zinc supplement along with a constant alcohol consumption. The present study demonstrates that a direct addition of zinc in the form of ZnGSH to Chinese Baijiu is feasible to accomplish the goal of zinc supplementation instantly along with alcohol consumption.

We made an effort to screen zinc complexes that do not change any of the properties of Chinese Baijiu on the consumer side but make zinc instantly available in the body for the reactions catalyzed by ADH and ALDH. ZnGSH thus fulfilled the requirement. The typical flavor characteristic indexes of ZnGSH-containing Chinese Baijiu were measured by a professional third-party testing institute in comparison with the original Chinese Baijiu. The results showed that there was no difference between the two formulations. To fulfill the appreciation of typical Chinese Baijiu drinkers, we invited professional Chinese Baijiu tasters to blindly compare the taste between Chinese Baijiu and Chinese Baijiu containing ZnGSH for any distinction, concluding no difference between the two.

The typical Chinese Baijiu caused a small percentage (6%) of death in mice at the dose used here, but this mortality was completely prevented, corresponding well with the shortened time to obtain sobriety in the mice dosed with Chinese Baijiu containing ZnGSH. This is a critical measure for the improvement of alcohol consumption on the drinker side. This significantly shortened time to obtain sobriety would greatly help drinkers recover from their drunkenness, avoiding much of the life-threatening effect of excessive alcohol drinking.

The analyses of plasma biomarkers of liver injury, including AST and ALT, revealed a typical acute liver damage after a single high gastric dose of Chinese Baijiu, mimicking a binge drinking condition in humans. As the result demonstrated that in the first 24 h, the enzyme activities of AST and ALT were significantly elevated as sampled at 12 and 24 h after dosing and basically returned to normal levels at 48 h after dosing. This early elevation of plasma AST and ALT was significantly diminished in the mice dosed with Chinese Baijiu containing ZnGSH. This protective effect of ZnGSH was further confirmed by the suppression of liver pathological changes induced by Chinese Baijiu, including hepatocyte steatosis, necrosis, inflammatory cell infiltration, and overall lipid deposition in the liver, following the same time-course changes in plasma AST and ALT. This suppression of liver pathological changes corresponded well to the reduction in acetaldehyde in the liver of mice treated with Chinese Baijiu containing ZnGSH. In addition, injury to the intestine by Chinese Baijiu, as previously demonstrated by ethanol exposure, including cellular swelling, apoptosis, gut leakiness and endotoxemia [[22], [23], [24], [25]], was also prevented by ZnGSH. These protective effects of ZnGSH in Chinese Baijiu on the liver and intestine thus proved the effectiveness of ZnGSH addition to Chinese Baijiu for the prevention of alcohol-induced multiple organ damages.

Alcohol is absorbed through the gastrointestinal tract into the blood circulation and is mainly metabolized by hepatocytes in the liver [26]. ADH and ALDH are two key enzymes in the metabolism of alcohol [27]. Zinc is a cofactor of ADH and ALDH, and the removal of zinc from ADH leads to a complete loss of its catalytic activity [5]. The prevention of alcohol-associated liver and intestinal injury most likely resulted from ZnGSH addition to increase alcohol metabolic enzymes (ADH and ALDH). The results showed that the ADH activity in the liver at 12 h was significantly increased, and subsequently, the ALDH at 24 h was significantly increased. A similar tendency was observed in the ALDH activity in the stomach at 24 and 48 h and in the intestine at 48 h. The increase in the ADH and ALDH activity in the liver was associated with the increase in zinc concentrations in the liver of mice treated with Chinese Baijiu containing ZnGSH. These overall effects would significantly decrease the burden of hepatic alcoholic metabolism and normalize alcoholic metabolism in the body as a whole.

It is important to note that Chinese Baijiu significantly decreased GSH concentrations in the liver at all time points measured, and ZnGSH in Chinese Baijiu not only prevented this depletion but also increased GSH concentrations in the liver. GSH is not only able to form a complex with zinc but itself also has an antioxidant capacity [9]. Physiologically, hepatic zinc and GSH regulatory systems balance their levels [[28], [29], [30]]. However, alcohol consumption impaired the antioxidant defense in the liver [9], increasing the demand for zinc and GSH in the liver to protect against damage. ZnGSH supplementation thus repletes the levels of Zn and GSH in the liver. GSH is the most important molecule involved in cellular antioxidant defense, and selective GSH depletion in mitochondria has been repeatedly reported in ethanol-exposed animals [9]. Coordination of zinc to the thiol group of cysteine residues and the low redox potential of the thiolate potential of the thiolate clusters make the coupling between zinc and redox metabolism possible [29]. It has been shown that changes in GSH modulate zinc transfer from zinc-binding proteins to others [29]. In this study, we chose the optimal dose of zinc as defined previously [10,14]. But we did not specify the optimal dose of GSH based upon the assumption that the use of GSH here only makes zinc more available, as reflected by the results. Due to the beneficial effect in the binge drinking model, we believe that the same beneficial effect of ZnGSH on individuals who chronically consume alcohol would be expected, which will be tested in future studies.

This study used Chinese Baijiu as a sample alcoholic beverage, but the application of ZnGSH to other alcoholic beverages would be predictably feasible. The solubility of the amount of ZnGSH needed for the observed preventive effect has been tested to be in the solution containing less than 60% alcohol (v/v, Chinese Baijiu here contained 53% alcohol). Therefore, the result here can be extrapolated to a majority of alcoholic beverages containing less than 60% alcohol.

In conclusion, the present study demonstrated that Chinese Baijiu containing ZnGSH did not alter the likeliness of its drikers but prevented the alcohol-associated liver injury in consumers. The hepatoprotective effect of ZnGSH on alcohol-associated liver injury most likely results from an increase in alcoholic metabolism, due to the increase in zinc availability along with alcohol consumption. These results suggest that the addition of ZnGSH to alcoholic beverages could make these beverage less tissue deteriorating and may have socioeconomic value in the prevention of alcohol-associated liver disease.

## Author contribution statement

Yinrui Feng: Performed the experiments; Analyzed and interpreted the data; Contributed reagents, materials, analysis tools or data; Wrote the paper.

Wenrui Liu, Yuan Ma, Guotao Tang: Performed the experiments; Analyzed and interpreted the data.

Te Ba, Zhenghui Luo: Contributed reagents, materials, analysis tools or data.

Y.James Kang: Conceived and designed the experiments; Wrote the paper.

## Funding statement

This work was supported by 10.13039/501100001809National Science Foundation of China [81230004].

## Data availability statement

Data will be made available on request.

## Declaration of interest's statement

The authors declare no conflict of interest.
